# Visual Blurring Following Posterior Vitreous Detachment in Fuchs Uveitis Syndrome

**DOI:** 10.18502/jovr.v21.18382

**Published:** 2026-07-13

**Authors:** Alireza Hedayatfar, Soon-Phaik Chee

**Affiliations:** ^1^Eye Research Center, The Five Senses Institute, Rassoul Akram Hospital, Iran University of Medical Sciences, Tehran, Iran; ^2^Noor Ophthalmology Research Center, Noor Eye Hospital, Tehran, Iran; ^3^Singapore National Eye Centre, Singapore Eye Research Institute, Singapore; ^4^Department of Ophthalmology, Yong Loo Lin School of Medicine, National University of Singapore, Singapore; ^5^Duke-NUS Medical School, Singapore

**Keywords:** Fuchs Uveitis Syndrome, Posterior Vitreous Detachment, Uveitis

## Abstract

**Purpose:**

To describe the clinical presentation, diagnostic challenges, management, and visual outcome of acute posterior vitreous detachment (PVD) in patients with Fuchs uveitis syndrome (FUS)

**Methods:**

This retrospective case series included consecutive patients with FUS who presented with acute PVD at a referral center from 2020 to 2024. The diagnosis of PVD was established based on fundus examination and dynamic B-scan ultrasonography findings.

**Results:**

Sixteen eyes of 16 patients (11 females, 5 males) with a mean age of 43 ± 9.8 years were studied. All patients reported sudden blurred vision in a white, painless eye, with 43.8% noting a new onset or an increased floater. The mean best-corrected visual acuity (BCVA) ranged from 20/25 to 20/200 at presentation, with an average of 0.43 logMAR. B-scan imaging revealed that following PVD, the majority of echogenic vitreous opacities were confined to the detached, shrunken vitreous body, and in some instances, accumulated within the peripheral vitreous cortex. The liquefied vitreous humor between the retina and the posterior hyaloid remained relatively echo-free. Within 3 months, spontaneous visual improvement occurred in 62.5% of patients, with mean BCVA improving from 0.310 to 0.127 logMAR. In three patients with persistent symptoms, pars plana vitrectomy (PPV) resulted in significant visual improvement, with the mean logMAR changing from 0.740 to 0.133.

**Conclusion:**

Acute PVD in patients with FUS can lead to sudden blurred vision, likely due to the increased density of inflammatory cells, opacities, and condensed vitreous strands within a detached, shrunken vitreous gel. In some cases, inflammatory deposits were layering over the peripheral vitreous body, contributing further to visual disturbance. Although most eyes showed spontaneous improvement, PPV was an effective intervention for patients with persistent, intolerable symptoms.

##  INTRODUCTION 

Fuchs uveitis syndrome (FUS) is a chronic, often unilateral, and low-grade uveitis characterized by diffuse iris stromal atrophy, stellate keratic precipitates (KPs), and the absence of posterior synechiae (PS) and cystoid macular edema (CME) with minimal associated redness or pain.^[1,2,3]^ The condition is often underdiagnosed due to its asymptomatic or insidious onset.^[4]^ FUS is frequently complicated by cataract and secondary glaucoma. Development of a unilateral posterior subcapsular cataract in a young patient may be the presenting symptom. Heterochromia is common in light-colored irises but less apparent in darker irises, leading to the preferred terminology “FUS” over “Fuchs heterochromic iridocyclitis”.^[1]^

The vitreous is frequently affected in FUS.^[2,5]^ These patients may experience floaters as a result of inflammatory deposits within the vitreous cavity. Prior surgeries and chronic low-grade inflammation may weaken the adhesion between the vitreous and retina, predisposing FUS patients to early vitreoretinal separation and posterior vitreous detachment (PVD).^[6]^ PVD may lead to noticeable visual disturbances in normal eyes, but due to the accumulation of inflammatory deposits in eyes with FUS, it can result in visual blurring.

Acute PVD in patients with FUS and its outcomes have not been well reported in the literature. In this retrospective case series of 16 patients, we study the clinical presentation, diagnostic challenges, management, and outcomes of acute PVD in patients with FUS.

##  METHODS

In this retrospective case series, we reviewed the medical records of consecutive patients with FUS who presented with a sudden onset of blurred vision due to acute PVD at Rasoul Akram Hospital between 2020 and 2024. The inclusion criteria were a confirmed diagnosis of FUS and evidence of acute PVD on funduscopy and dynamic B-scan ultrasonography. Cases with vitritis but no PVD were excluded. The study was conducted in accordance with the Declaration of Helsinki and was approved by Research Ethics Committee of Iran University of Medical Sciences.

The collected data included patient demographics, visual acuity, slit lamp examination, and posterior segment findings. The investigation results consisted of dynamic B-scan ultrasonography, optical coherence tomography (OCT) assessment of macula and optic nerve head, wide-field fluorescein angiography (FA), and, when necessary, targeted lab and polymerase chain reaction (PCR) of ocular fluid to exclude other possible etiologies.

FUS diagnosis was mostly based on clinical findings as previously described in the literature, including low-grade anterior uveitis, diffuse iris atrophy, diffuse stellate KPs, absence of PS, and lack of CME.^[1,2,3]^

The occurrence of PVD was established based on a combination of clinical observations and corroborative findings from B-scan ultrasonography. During the fundus examination with a 90-diopter lens, even in conditions of suboptimal media clarity, we were often able to identify either a well-defined Weiss ring or a recognizable detached posterior hyaloid face. This detachment created a clear space between the cloudy vitreous body and the retinal surface.

Dynamic B-scan ultrasonography was performed in selected cases, particularly in eyes with severe media opacity. This evaluation involved assessing reflectivity and after-movement patterns (to differentiate between PVD and retinal detachment), as well as identifying the remaining attachment of the vitreous body to the optic disc and peripheral retina. Complete PVD was characterized by a low- to medium-reflective membrane that was entirely separated from the retina, often exhibiting after movement. In contrast, incomplete PVD was characterized by a partially detached reflective membrane that still had some attachments to the optic disc and/or peripheral retina.

Patients were monitored at 2 weeks, 1 month, and 3 months. Surgery was considered only if vision failed to improve within 3 months. Since the guidelines for performing vitrectomy in this situation are not well established, we based our decision mainly on the persistence and severity of the patients' symptoms. We also considered factors such as the patients' previous visual acuity, whether they were phakic or pseudophakic, and the presence of any accompanying cataracts. For those selected for surgery, a standard 23-gauge pars plana vitrectomy (PPV) was performed.

Statistical analysis was performed using R Statistical Software, with results presented as mean ± standard deviation for continuous variables and frequencies for categorical variables.

##  RESULTS

Sixteen eyes from 16 patients with a mean age of 43 ± 9.8 years (range, 37 to 68 years) were included. There was a female predominance (11, 68.8%), and the majority (15, 93.8%) were unilateral. In the patient with bilateral FUS, only the eye affected by acute PVD was included. Thirteen patients (81.3%) had been previously diagnosed with FUS, while three were new cases. All patients reported a sudden onset of blurred vision without pain or redness. Seven patients reported that the blurred vision was associated with an increase in their existing floaters (five patients) or the onset of new floaters (two patients). The median time from symptom onset to examination was 7 days (range, 3 days to 6 months).

Seven patients had previously undergone cataract surgery (median: 3 years ago, range: 1-15 years). Of these patients, four had received YAG laser capsulotomy. Additionally, five eyes had a history of glaucoma, and two of those eyes had undergone Ahmed glaucoma valve surgery. Four eyes were being treated with pressure-lowering eye drops. One patient had previously had myopic LASIK surgery.

At presentation, the best-corrected visual acuity (BCVA) ranged from 20/25 to 20/200 with an average of 0.43 logMAR (logarithm of the minimum angle of resolution). All eyes exhibited mild anterior chamber cells ( ≤ 2+ in all cases).^[7]^ Fifteen eyes (93.8%) presented with diffuse small- to medium-sized KPs, which appeared round or stellate in shape and were white or translucent in color. Diffuse iris stromal atrophy was noted in 13 eyes (81.3%). In 10 eyes (62.5%), there were white to yellowish mini iris nodules at the pupil, but no PS was present. None of the eyes showed heterochromia. Variable intensity of vitreous cells, opacities, and condensed vitreous strands was recorded in all cases. During the fundus examination, evidence of PVD was detectable in all but one eye due to hazy media (incomplete PVD was confirmed on B-scan ultrasonography). No retinal tears or detachments were observed. Glaucomatous optic disc changes were noted in five eyes.

B-scan ultrasonography was performed on 12 eyes, revealing complete PVD in five and incomplete PVD in seven patients. In each case, various degrees of echogenic spots were observed, localized to the detached vitreous gel, with a relatively echo-free space surrounding it. Additionally, a thick, irregular, echogenic membrane in the peripheral vitreous body was detected in eight patients. The retina remained attached in all cases.

Macular SD-OCT (performed in 17 eyes) demonstrated a normal inner and outer retina structure with no evidence of macular edema. A fine ERM was identified in one patient. Peripapillary SD-OCT (conducted in 12 eyes) documented varying degrees of nerve fiber layer loss in seven eyes.

Seven patients underwent FA. An indistinct amoeboid (five eyes) or a round, central, hypofluorescent shadow (two eyes) was observed, corresponding to PVD blocking the background fluorescence. A hyperfluorescent disc was detectable in five eyes, while two eyes demonstrated limited peripheral vascular leakage. None of the eyes exhibited macular leakage.

If the clinical presentation indicated a diagnosis of FUS, additional laboratory tests were usually not ordered. In this series, three patients with uncharacteristic clinical features (e.g., bilateral involvement) underwent blood tests and chest X-ray or CT scans to rule out other infectious and noninfectious etiologies. For one eye (case 3), in addition to laboratory tests, aqueous PCR was performed, yielding negative results for HSV, VZV, CMV, and toxoplasmosis.

Within 3 months, symptoms improved spontaneously in 10 patients (62.5%), with their BCVA increasing by 1 to 3 lines (mean logMAR improved from 0.310 to 0.127). Six patients reported no change or minimal improvement in their vision. Three of these six patients underwent a standard PPV. Visual acuity improved by three to five lines, and the mean logMAR reached from 0.740 to 0.133. One patient had a combined cataract and PPV surgery; her vision improved from 20/200 to 20/25.

Three cases with different scenarios are described below:

### Case 1

A 30-year-old man, diagnosed with FUS 5 years ago, reported experiencing photopsia and blurred vision in his right eye over the past week. These symptoms were not accompanied by pain or redness. BCVA was 20/200 compared to 20/25 during his previous clinic visit 4 months earlier. The left eye was amblyopic due to high astigmatism.

**Figure 1:**
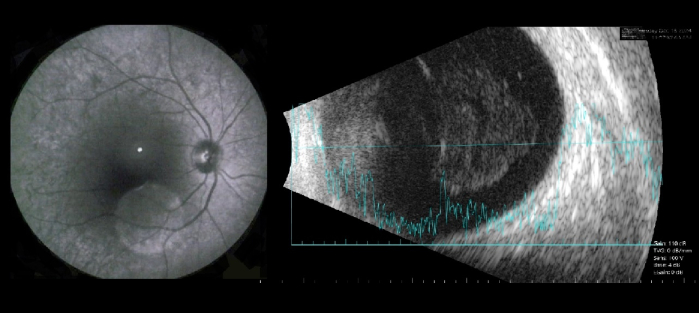
Left: The infrared image of the fundus reveals the central shadow of a detached vitreous body. Right: B-scan ultrasonography showing complete posterior vitreous detachment (PVD) with a shrunken vitreous body containing numerous fine echogenic opacities (gain: 110 dB).

Slit-lamp examination of the right eye revealed 0.5+ anterior chamber cells, diffuse stellate KPs, and diffuse iris stromal atrophy without transillumination defect or PS. The crystalline lens was clear, but the retrolental space appeared densely filled with cells and opacities. Funduscopic examination showed hazy media with a Weiss ring in the mid-vitreous cavity. The retina was attached, with no chorioretinal scars. The optic disc was not swollen, with a cup-to-disc ratio of about 0.5. The infrared image of the fundus reveals the central shadow of a detached vitreous body. B-scan ultrasonography confirmed the presence of a complete PVD with a shrunken vitreous body containing numerous fine echogenic opacities [Figure 1].

The patient was advised to be vigilant for signs of retinal detachment and scheduled close follow-up appointments. At the 3-month follow-up, the photopsia had resolved, but his visual acuity remained at 20/40. He was offered the option of surgery or continued follow-up. The benefits and risks of PPV were explained, in particular, the risk of developing cataract. He chose to postpone surgery.

### Case 2

A 43-year-old woman presented with a sudden, painless blurring of vision that had occurred 2 weeks prior in the left eye. She had previously been diagnosed with FUS and had undergone uncomplicated cataract surgery with intraocular lens implantation 3 years earlier, followed by YAG laser capsulotomy 1 year later. At her last visit 9 months earlier, BCVA had been 20/25 in the left eye, but it deteriorated to 20/40.

**Figure 2:**
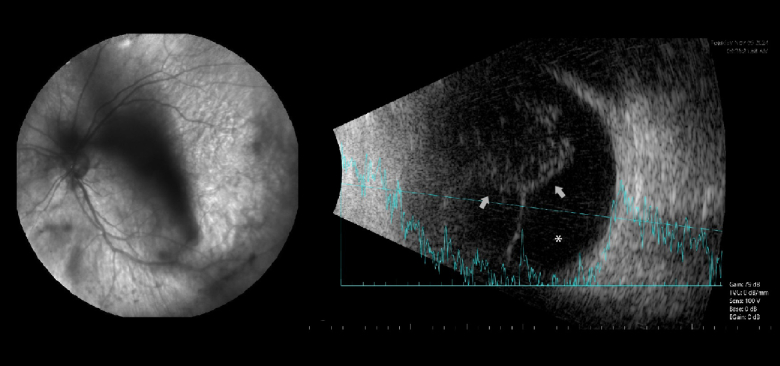
Left: The infrared image of the fundus reveals the shadow of the partially detached vitreous body over the macula. Right: B-scan ultrasonography (gain: 79 dB) demonstrates a detached vitreous body that retains attachments to the peripheral retina (incomplete PVD). The echogenic opacities are mainly confined to the thickened, irregular peripheral vitreous cortex (arrows). Note the echo-free space between the detached vitreous body and the retina (asterisk).

The slit-lamp examination of the left eye showed trace cells but no flare in the anterior chamber and scarce translucent round or stellate KPs speckled on the corneal endothelium. Several small, fluffy iris nodules were visible around the pupil with no PS. The vitreous contained clusters of cells attached to condensed strands of vitreous. The retina was normal, and OCT showed no macular edema. Direct funduscopy using a 90-diopter lens revealed a detached posterior hyaloid face. B-scan ultrasonography demonstrated an incomplete PVD, with a thickened peripheral vitreous cortex contributing significantly to the echogenic opacity. This might imply the accumulation of inflammatory cells, opacities, and condensed vitreous strands on the peripheral vitreous cortex [Figure 2]. Three months later, she continued to experience unstable, blurred vision and disturbing floaters. Due to the lack of improvement, PPV was performed, restoring the vision to 20/20.

### Case 3 

A 47-year-old woman with controlled hypertension was referred for treatment of panuveitis, which was refractory to previous therapies. She had experienced painless blurring of vision in her right eye 2 months earlier. Despite being treated with systemic and topical corticosteroids, the inflammation did not improve.

At the time of examination, BCVA was 20/40, and there was no relative afferent pupillary defect. IOP measured 26 mmHg on timolol and dorzolamide eye drops. There were 1+ cells in the anterior chamber, but KPs and PS were absent. Diffuse iris atrophy and moderate PSC cataracts were evident. The vitreous body contained cells and opacities, with a clear area behind the detached posterior hyaloid face. Retinitis and vasculitis were not present. The optic disc appeared pink and sharp, with a cup-to-disc ratio of approximately 0.7

**Figure 3:**
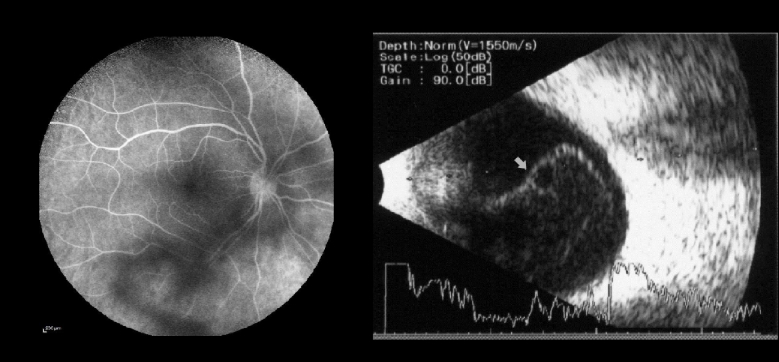
Left: Fluorescein angiography during the arteriovenous phase shows a hot disc and hypofluorescent amoeboid shadow of the detached vitreous body. Right: B-scan ultrasonography (gain: 90 dB) shows the detached vitreous gel encompassing numerous fine reflective opacities and a thickened echogenic membrane in the peripheral vitreous compartment (arrow).

B-scan ultrasonography revealed an incomplete PVD with numerous fine reflective opacities within the vitreous gel as well as a thick echogenic membrane lining the peripheral vitreous body. FA showed a hot disc without any perivascular or macular leakage. A large, indistinct, amoeboid hypofluorescent shadow obscured the typical fluorescence of the posterior pole [Figure 3]. OCT confirmed the absence of CME.

We diagnosed FUS complicated by PVD in the right eye and discontinued both the systemic and topical corticosteroids, recommending close follow-up instead. One month later, faint diffuse stellate KPs appeared, which supported our diagnosis. Without further intervention, at the 3-month follow-up, BCVA improved to 20/25.

##  DISCUSSION

The findings of this case series highlight acute PVD as an underrecognized cause of sudden blurred vision in patients with FUS. Following PVD, the accumulation of inflammatory cells, opacities, and condensed vitreous strands within the detached and shrunken vitreous body, as well as a layering of inflammatory deposits over the peripheral vitreous cortex, can lead to significant visual disturbances and blurred vision.

PVD is a common age-related condition that occurs as the vitreous gel separates from the retina, typically following progressive vitreous liquefaction (synchysis) and the aggregation of collagen fibrils (syneresis).^[8,9]^ While often associated with aging, PVD can also arise in younger individuals due to trauma, surgery, or chronic eye conditions.^[9]^ In FUS, chronic inflammation and structural changes within the vitreous predispose patients to early PVD.^[6]^ Moreover, prior cataract and glaucoma surgeries, which are common in this population, may further contribute to early vitreous degeneration and PVD formation.^[10,11]^

The vitreous is frequently affected in FUS, with several studies reporting the presence of inflammatory cells and deposits within the vitreous cavity, which manifest as floaters.^[2,4]^ These opacities are usually mild and well-tolerated by most patients.^[4]^ In this study, all patients who experienced acute PVD reported a sudden onset of blurred vision. Notably, 43.8% patients indicated that this visual disturbance was accompanied by new floaters or a noticeable increase in pre-existing floaters. These symptoms were qualitatively different from the subtle and often less perceptible floaters typically associated with FUS. In contrast to age-related PVD, which rarely causes a decline in visual acuity, the acute presentations observed here were more visually significant.

**Figure 4:**
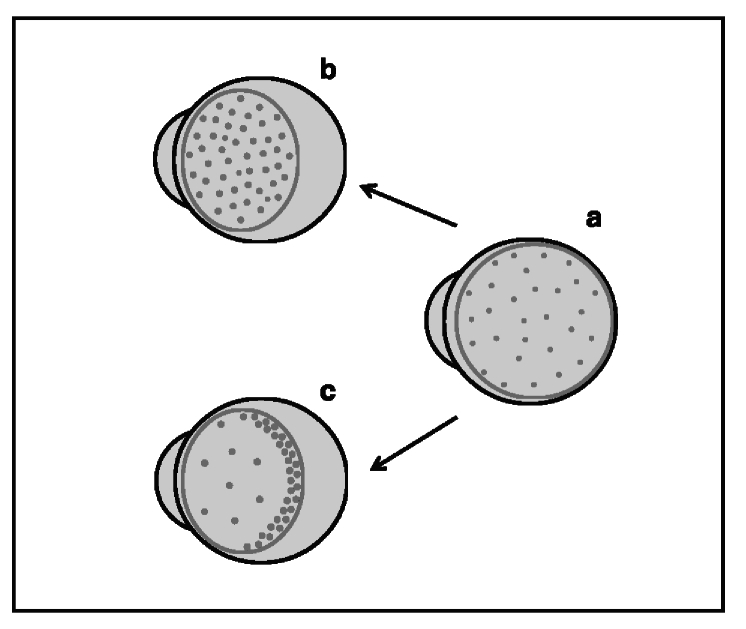
Schematic illustration of a proposed mechanism contributing to visual disturbance in Fuchs Uveitis Syndrome following posterior vitreous detachment (PVD). (a) Inflammatory opacities were diffusely distributed in the vitreous before PVD. (b) Increased density of vitreous opacities following PVD. (c) Layering of inflammatory opacities over the peripheral vitreous cortex.

B-scan imaging revealed that the majority of inflammatory cells, opacities, and condensed vitreous strands following PVD were located within the detached vitreous compartment, leaving the liquefied vitreous humor between the retina and the posterior hyaloid face relatively echo-free. The concentration of cloudy material, normally dispersed throughout the entire vitreous cavity, now increases within the reduced volume of the detached and shrunken vitreous body [Figure 4b]. This elevated concentration leads to greater light scattering, resulting in sudden blurred vision and a more pronounced sensation of floaters. Additionally, inflammatory cells, opacities, and condensed vitreous strands may accumulate along the peripheral vitreous cortex, transforming this normally transparent interface into a partially opaque membrane, thereby contributing further to visual disturbance [Figure 4c].

In our case series, most patients experienced a spontaneous reduction in symptoms over time. Blurred vision improved in 10 out of 16 patients, and the floaters either returned to baseline levels, similar to those experienced before the acute event, or became less noticeable. The exact mechanisms behind the improvement of symptoms over time, despite the structural opacities remaining visible, are not entirely understood. It is possible that the opacities, which were initially concentrated in the detached vitreous gel near the posterior pole, gradually shift away from the visual axis, reducing their optical impact. Additionally, neuroadaptation may play a role in making the floaters less perceptible. This emphasizes the importance of recognizing the benign nature of this condition and effectively communicating to patients the possibility of spontaneous symptom improvement. Therefore, it is advisable to avoid rushing into early surgery. However, clinicians should inform patients about the warning signs of retinal detachment and schedule frequent follow-up appointments.

Technological advances in both instrumentation and techniques have made PPV a much safer procedure.^[12]^ Small-gauge vitrectomy is a minimally invasive and often well-tolerated method for removing symptomatic floaters.^[13]^ With careful patient selection, the benefits of surgical intervention can greatly outweigh the risks ^[14]^. In our study, for patients who experienced persistent and significant blurred vision or bothersome floaters, a standard three-port, 23-gauge PPV effectively relieved symptoms and restored vision. No significant complications were observed, except for a mild fibrin reaction in one case postoperatively. Additionally, smaller-gauge vitrectomy may serve as a commendable alternative in similar cases, due to reduced postoperative inflammation and a quicker recovery, depending on the surgeon's preference and the case's complexity.

This study is limited by its retrospective design and small sample size, underscoring the need for further research to elucidate the mechanisms underlying symptomatic PVD in patients with FUS. Due to the study's retrospective nature, not all patients underwent B-scan evaluations before presenting with acute symptoms. Therefore, a pre- and post-study of B-scan changes was not feasible for most cases. Additionally, long-term follow-up is needed to determine whether the floaters and opacities recur.

In summary, acute PVD in patients with FUS can lead to significant visual disturbances and blurred vision. In most cases, symptoms tend to improve over time, though PPV effectively restores vision in selected cases. Proper diagnosis and patient counseling are essential to avoid unnecessary interventions.

##  Financial Support and Sponsorship

None.

##  Conflict of Interest 

None.
